# Life without complex I: proteome analyses of an Arabidopsis mutant lacking the mitochondrial NADH dehydrogenase complex

**DOI:** 10.1093/jxb/erw165

**Published:** 2016-04-27

**Authors:** Steffanie Fromm, Jennifer Senkler, Holger Eubel, Christoph Peterhänsel, Hans-Peter Braun

**Affiliations:** ^1^Institut für Pflanzengenetik, Leibniz Universität Hannover, Herrenhäuser Str. 2, 30419 Hannover, Germany; ^2^Institut für Botanik, Leibniz Universität Hannover, Herrenhäuser Str. 2, 30419 Hannover, Germany

**Keywords:** *Arabidopsis thaliana*, carbonic anhydrase, complex I, mitochondrial metabolism, photosynthesis, proteomics, respiratory chain.

## Abstract

The mitochondrial NADH dehydrogenase complex (complex I) is of particular importance for the respiratory chain in mitochondria. It is the major electron entry site for the mitochondrial electron transport chain (mETC) and therefore of great significance for mitochondrial ATP generation. We recently described an *Arabidopsis thaliana* double-mutant lacking the genes encoding the carbonic anhydrases *CA1* and *CA2*, which both form part of a plant-specific ‘carbonic anhydrase domain’ of mitochondrial complex I. The mutant lacks complex I completely. Here we report extended analyses for systematically characterizing the proteome of the *ca1ca2* mutant. Using various proteomic tools, we show that lack of complex I causes reorganization of the cellular respiration system. Reduced electron entry into the respiratory chain at the first segment of the mETC leads to induction of complexes II and IV as well as alternative oxidase. Increased electron entry at later segments of the mETC requires an increase in oxidation of organic substrates. This is reflected by higher abundance of proteins involved in glycolysis, the tricarboxylic acid cycle and branched-chain amino acid catabolism. Proteins involved in the light reaction of photosynthesis, the Calvin cycle, tetrapyrrole biosynthesis, and photorespiration are clearly reduced, contributing to the significant delay in growth and development of the double-mutant. Finally, enzymes involved in defense against reactive oxygen species and stress symptoms are much induced. These together with previously reported insights into the function of plant complex I, which were obtained by analysing other complex I mutants, are integrated in order to comprehensively describe ‘life without complex I’.

## Highlight

Complex I dysfunction causes reorganization of cellular respiration and affects metabolic processes in mitochondria, plastids, peroxisomes, and other cellular compartments with drastic consequences for growth and development.

## Introduction

Cellular respiration is the fundamental ATP generating process common to most eukaryotes. Mitochondria carry out the final steps of this process and efficiently generate ATP through oxidative phosphorylation (OXPHOS). The mitochondrial OXPHOS system consists of five inner mitochondrial membrane-embedded protein complexes – the four respiratory chain protein complexes (complexes I–IV) and the ATP synthase complex (complex V) – and two mobile electron transporters (ubiquinone and cytochrome *c*).

The OXPHOS complexes catalyse the electron transfer from NADH or FADH_2_ to molecular oxygen as the terminal electron acceptor. Electrons are inserted into the mitochondrial electron transport chain (mETC) via NADH generated by glycolysis, the TCA cycle (additionally electrons come from FADH_2_), and other catabolic processes such as the photorespiration pathway in plants. As first proposed by Peter Mitchell (1961), electron transport at the mETC is coupled to the translocation of protons from the mitochondrial matrix into the intermembrane space. This creates an electrochemical gradient across the inner mitochondrial membrane that results in a proton motive force. Complex V can use this proton gradient to generate ATP, which finally is exported and used for driving energy-demanding processes.

The NADH dehydrogenase complex (complex I) is of special importance for the OXPHOS system because it is the main site for electron insertion into the mETC and can provide up to 40% of the protons for mitochondrial ATP formation (Hunte *et al.*, 2010; Braun *et al.*, 2014). The structure of complex I has been investigated in *Escherichia coli*, *Thermus thermophilus*, *Bos taurus*, and *Yarrowia lipolytica* (Morgan and Sazanov, 2008; Baradaran *et al.*, 2013; Vinothkumar *et al.*, 2014; Zickermann *et al.*, 2015). Its L-like shape, which originates from two orthogonally arranged ‘arms’, is well conserved in these species. One arm is hydrophobic, embedded in the inner mitochondrial membrane, and termed the ‘membrane arm’. The second arm, which is designated the ‘peripheral arm’, is hydrophilic and is attached to the end of the membrane arm. It protrudes into the mitochondrial matrix. In plants, complex I contains an additional spherical domain, which is attached to the membrane arm at a central position on its matrix-exposed side (Dudkina *et al.*, 2005; Sunderhaus *et al.*, 2006).

Despite overall similarity in shape, complex I from prokaryotes is comparatively small and has a simple subunit composition. The 14 subunits present in the 550kDa complex I of *E. coli* constitute the ‘minimal set’ of subunits (Weidner *et al.*, 1993; Yagi and Matsuno-Yagi, 2003; Sazanov, 2007). Complex I of eukaryotes is nearly twice as large (1000kDa) (Friedrich and Böttcher, 2004; Brandt, 2006). In addition to the conserved ‘minimal set’ of subunits, eukaryotic complex I includes several accessory subunits (Friedrich, 2001). However, due to the occurrence of some lineage-specific accessory subunits the overall number of complex I subunits varies between different eukaryotes (e.g. *Chlamydomonas reinhardtii*: 42; *Yarrowia lipolytica*: 42; *Bos taurus*: 45; *Arabidopsis thaliana*: 49) (Cardol *et al.*, 2004; Carroll *et al.*, 2006; Angerer *et al.*, 2011; Peters *et al.*, 2013).

Plant complex I includes nine lineage-specific subunits (Braun *et al.*, 2014), most notably a group of carbonic anhydrases located within the spherical extra domain attached to the membrane arm (Sunderhaus *et al.*, 2006). Within this domain, which is designated the ‘carbonic anhydrase domain’ of plant complex I, three gamma-type carbonic anhydrase (γCA) proteins (γCA1, γCA2, γCA3) and two gamma-type carbonic anhydrase-like (γCAL) proteins (γCAL1 and γCAL2) are located (Perales *et al.*, 2004; Klodmann *et al.*, 2010). Since the γCA domain has a molecular mass of 85kDa it can only include three out of the five γCA/γCAL proteins at the same time (Klodmann *et al.*, 2010). Based on investigations using γCA/γCAL mutants, six possible subunit arrangements have been suggested to occur (Fromm *et al.*, 2016a): each γCA domain contains either the γCAL1 or the γCAL2 protein and additionally two copies of the γCA proteins (γCA1 or γCA2, or both but not γCA3).

The γCA/γCAL subunits have been found to be essential for the early steps of complex I assembly (Meyer *et al.*, 2011; Li *et al.*, 2013). Deletion of the gene encoding Arabidopsis γCA2 causes reduction of complex I (Perales *et al.*, 2005). Deletion or downregulation of more than one gene encoding the γCA/γCAL subunits in Arabidopsis has drastic effects on the amount of complex I (Soto *et al.*, 2015; Fromm et al., 2016b, c). Similarly, deletion of other genes encoding complex I subunits has been reported to cause significant reduction of complex I levels (e.g. deletion of the *ndufs4*, *bir6*, and *slo3* genes; Kühn *et al.*, 2015; Koprivova *et al.*, 2010; Hsieh *et al.*, 2015). In contrast to vertebrates, plants can withstand complex I reduction and dysfunction because they possess alternative NADH dehydrogenases in the mitochondria, which are important for mitochondrial functioning, especially if plants are growing under unfavorable growth conditions (Rasmusson *et al.*, 2008).

A few Arabidopsis mutants have been described that completely lack mitochondrial complex I. In one mutant the NDUFV1 subunit (also called the 51-kDa subunit of complex I) is missing, causing complete absence of complex I. The mutant displayed increased fluxes through glycolysis and the TCA cycle (Kühn *et al.*, 2015). In a second complex I-deficient Arabidopsis mutant the genes encoding γCA1 and γCA2 are absent. Mutant seeds do not germinate but can be rescued in the presence of high sucrose by an embryo rescue method (Fromm *et al.*, 2016c). Growth of the resulting plants is extremely retarded. In the *ca1ca2* mutant the cytochrome *c* oxidase complex is much induced, probably compensating for the loss of complex I with respect to proton translocation across the inner mitochondrial membrane (Fromm *et al.*, 2016c).

Complex I-deficient mutants offer the unique opportunity to study ‘plant life without complex I’. Here we report a systematic proteomic comparison between *ca1ca2* and wildtype Arabidopsis lines that is based on three distinct experimental strategies: 2D IEF/SDS PAGE, 2D BN/SDS PAGE, and a label-free quantitative shotgun proteome approach. MS analyses allowed in-depth insights into the molecular mechanisms compensating for the lack of complex I.

## Material and Methods

### Plant material and growth conditions

Arabidopsis (*Arabidopsis thaliana*) lines used for this study were of the Columbia ecotype. The SALK_109391 (AT1G19580, *ca1/ca1*) and SALK_010194 (AT1G47260, *ca2/ca2*) mutant lines were obtained from The European Arabidopsis Stock Centre (NASC; Loughborough, UK). *ca1* and *ca2* single mutants were crossed to generate *ca1ca2* double-mutants (Fromm *et al.*, 2016c). Plants were grown on 0.5 Murashige and Skoog medium in climate chambers under the following conditions: 8h of light (120 µmol s^−1^ m^−2^)/16h of dark, 22 °C, 65% humidity, atmospheric CO_2_ concentrations. Homozygous *ca1ca2* mutants were germinated and rescued by cultivation on 0.5 MS medium containing 3% (w/v) sucrose. After 6 weeks plants were transferred to soil and cultivation was continued under the same conditions without sucrose. Wildtype and double-mutant plants were harvested at the 10-rosette-leaf developmental stage, and leaves were used for proteomic analyses.

Cell cultures of Arabidopsis lines were established as described by May and Leaver (1993). Callus was maintained as suspension culture according to Sunderhaus *et al.* (2006).

### Isolation of mitochondria

Mitochondria from cell culture were purified by differential centrifugation and Percoll density gradient centrifugation as described by Werhahn *et al.* (2001).

### Protein gel electrophoresis procedures and staining procedures

One-dimensional Blue Native PAGE (1D BN PAGE) was performed according to Wittig *et al.* (2006). Mitochondrial membranes were solubilized by digitonin at a concentration of 5g g^–1^ mitochondrial protein (Eubel *et al.*, 2003). For subsequent SDS PAGE, BN lanes with separated protein complexes were transferred horizontally onto SDS gels. Second-dimension PAGE was carried out as outlined previously (Wittig *et al.*, 2006). Differential gel electrophoresis (DIGE), which is based on labeling of proteins with CyDyes before 2D BN/SDS PAGE, was carried out according to Peters and Braun (2012).

Two-dimensional IEF/SDS PAGE was carried out as described by Mihr and Braun (2003). For the IEF gel dimension, Immobiline DryStrip gels (24cm, non-linear gradient pH 3–11) were used. Focusing took place for 24h at 30 to 8000V using the Ettan IPGphor 3 system (GE Healthcare).

For the second gel dimension, IPG stripes were equilibrated for 15min with DTT (0.4g/40ml) and then 15min with iodoacetamide (1.0g/40ml). SDS PAGE was carried out using the High Performance Electrophoresis (HPE) FlatTop Tower-System (Serva Electrophoresis) using precast Tris-Glycine gels (12.5% polyacrylamide, 24 x 20cm).

Gels were fixed for 2h in 15% (v/v) ethanol, 10% (v/v) acetic acid and stained with Coomassie Brilliant Blue G250 (Neuhoff *et al.*, 1985, 1990).

Comparative proteome analyses were based on gel triplicates and data evaluation using the Delta 2D software 4.3 (Decodon, Greifswald, Germany) according to Berth *et al.* (2007) and Lorenz *et al.* (2014).

### Protein identification by mass spectrometry

Tryptic digestion of proteins and their identification by mass spectrometry (MS) were performed as described by Klodmann *et al.* (2010). Peptide separation was carried out by using the EASY-nLC System (Proxeon, Thermo Scientific, Bremen, Germany) and coupled MS analyses by using the MicrOTOF-Q II mass spectrometer (Bruker Bremen, Germany). MS primary data were evaluated using the Proteinscape software package (version 2.1, Bruker, Bremen, Germany), the Mascot Search Engine (Matrix Science, London, UK), the Arabidopsis protein database (www.arabidopsis.org; release TAIR10), and an updated version of a complex I database (Klodmann *et al.*, 2010) that represents a subset of the Arabidopsis TAIR10 database. The threshold Mascot Score was set to 30 or 60 for proteins and 20 for peptides.

### Label-free quantitative shotgun mass spectrometry

#### Sample preparation for ESI-MS/MS

Total proteins of five biological replicates of wt and *ca1ca2* leaves were extracted. Then 50 µg of protein were solubilized in 2× sample buffer [4% (w/v) SDS, 125mM Tris-HCl (pH 6.8), 20% (v/v) glycerol, and 0.5% (w/v) bromophenol blue (BPB)] and loaded on a glycine/SDS gel [10% (w/v) acrylamide in stacking gel, 14% (w/v) in separation phase]. To concentrate proteins in a single band the gel run was stopped when the BPB front reached the end of the stacking gel. Gels were then Coommassie-stained and the protein bands were extracted and transferred into low-binding Eppendorf caps (Eppendorf, Wesseling-Berzdorf, Germany). After drying in a vacuum centrifuge (Eppendorf, Wesseling-Berzdorf, Germany), gel pieces were rehydrated in 200 µl reduction solution [20mM DTT, 0.1M ammonium bicarbonate (AmBiC)] for 30min at 56 °C. Afterwards, they were dehydrated again by addition of 200 µl acetonitrile (ACN) for 10min. The supernatant was removed and alkylation of cysteine residues was achieved by incubation in 200 µl alkylating solution (55mM iodoacteamide, 0.1M AmBiC) for 30min in the dark. After ACN-dehydration for 10min the supernatant was removed and 200 µl of 0.1M AmBiC were added. After 15min of incubation the supernatant was removed and gel pieces were dehydrated by addition of ACN. After removal of residual ACN, gel pieces were dried by vacuum centrifugation for 20min. The dried gel pieces were treated with trypsin (Promega, Mannheim, Germany) solution prepared according to the manufacturer’s instruction. Eighty microliters were added to each sample, which were subsequently incubated overnight at 37 °C. Extraction of peptides was initiated by adding 40 µl of 50% (v/v) ACN, 5% (v/v) formic acid (FA) (30min, 37 °C, 800rpm). The tryptic peptide-containing supernatants were collected in new low-binding Eppendorf tubes. The procedure was repeated twice by first adding 40 µl of 50% (v/v) ACN, 1% (v/v) FA, and then 100% (v/v) ACN afterwards. The supernatants for each sample were pooled in the same Eppendorf tubes and subsequently dried using a vacuum centrifuge at 30 °C. For mass spectrometry peptides were absorbed in 20 µl 2% (v/v) ACN, 0.1% (v/v) FA.

#### ESI-MS/MS

Tandem mass spectrometry (MS/MS) analysis was performed by means of a Q-Exactive (Thermo Fisher Scientific, Dreieich, Germany) mass spectrometer coupled to an Ultimate 3000 (Thermo Fisher Scientific, Dreieich, Germany) UPLC.

Seven microliters of sample solution were drawn from 0.25-ml glass insert vials (Sun-SRI, Rockwood, TN, US) kept at 8 °C in the sample compartment and stored in a 20-µl sample loop before being injected into a 2cm, C18, 5 µm, 100 Å reverse phase trapping column (Acclaim PepMap100, Thermo Fisher Scientific, Dreieich, Germany) at a rate of 4 µl min^–1^. Peptide separation was achieved on a 50cm, C18, 3 µm, 100 Å reverse phase analytical column (Acclaim PepMap100, Thermo Fisher Scientific, Dreieich, Germany). Peptides were eluted using a non-linear 2% to 30% (v/v) acetonitrile gradient in 0.1% (v/v) formic acid with a flow of 300 nl min^–1^ over a period of 60 mins and at a set column oven temperature of 35 °C. To clean the column, the ACN concentration was subsequently raised to 95% (v/v) within 10min, where it was kept for another 15min before column equilibration to 2% (v/v) ACN commenced.

Transfer of eluted peptides into the mass spectrometer was achieved by means of a NSI source (Thermo Fisher Scientific, Dreieich, Germany) using stainless steel nano-bore emitters (Thermo Fisher Scientific, Dreieich, Germany) connected to the column outlet by a 50-cm, 0.05mm ID fused silica capillary. During MS analysis spray voltage was set to 2.2kV, capillary temperature to 275 °C, and S-lens RF level to 50%. The MS was run in positive ion mode, MS/MS spectra (top 10) were recorded from 30 mins to 220min. For full MS scans, the number of microscans was set to 1, resolution to 70 000, AGC target to 1e6, maximum injection time to 400ms, number of scan ranges to 1, scan range to 400–1600 m/z, and spectrum data type to ‘profile’. For dd-MS2, the number of microscans was set to 1, resolution to 17 500, AGC target to 1e5, maximum injection time to 250ms, Loop count to 10, MSX count to 1, isolation window to 3.0 m/z, fixed first mass to 100.0 m/z, NCE to 27.0 (stepped NCE deactivated), and spectrum data type to ‘profile’. Data dependent (dd) settings were as follows: underfill ratio, 0.5%; intensity threshold, 2.0e3; apex trigger, 10 to 40s; charge exclusion, unassigned, 1, 5, 5–8, >8; peptide match, preferred; exclude isotopes, on; dynamic exclusion, 45.0s.

MS/MS data were queried against an in-house TAIR10 database, modified to also include common contaminants (keratin, trypsin), MS-standards (BSA, fibrinopeptide) and known modifications of mitochondrial encoded proteins based on RNA-editing (AGIs) using Proteome Discoverer (Thermo Fisher Scientific, Dreieich, Germany). Search runs employed the Mascot (Matrix Science, London, United Kingdom), peptide selector settings employed the following spectrum properties filter: Lower Rt limit, 0; upper RT limit, 0; first scan, 0; last scan, 0; lowest charge state, 1; highest charge state, 5; min. precursor mass, 350Da; max. precursor mass, 5000Da; total intensity threshold, 0; and minimum peak count, 1. The scan event filter was adjusted to the following settings: mass analyser, ftms; ms order, MS2; activation type, HCD; min. collision energy, 0; max. collision energy, 1000; scan type, full; ionization source, nanospray; polarity mode, +. The S/N threshold was set to 1.5. For Mascot, the number of maximum missed cleavage sites was limited to 1, precursor mass tolerance to 10 ppm, and fragment mass tolerance to 0.8Da. Allowed variable modifications were oxidation of methionine residues and N-terminal acetylations. Carbamidomethylation of cysteine residues was selected as fixed modification. For the target decoy PSM validator, strict target FDR was set to 0.01, while 0.05 was selected for relaxed target FDR.

#### Identification and protein quantification

Q-Exactive raw-files were loaded into the MaxQuant software (Cox and Mann, 2008) and processed using the following group specific parameters: variable modifications, acetyl (N-term), oxidation (M); digestion mode, specific; enzyme, Trypsin/P; max. number of missed cleavages, 2; match type, match from and to; number of threads, 3; max. instrument type, Orbitrap; first search peptide tolerance, 20; main search tolerance, 4.5; peptide tolerance unit, ppm; individual peptide mass tolerance, chosen; isotope match tolerance, 2 (ppm); centroid match tolerance, 8 (ppm); centroid half width, 35 (ppm); time valley factor, 1.4; isotope time correlation, 0.6; theoretical isotope correlation, 0.6; recalibration unit, ppm; use MS1 centroids, not chosen; use MS2 centroids, not chosen; intensity dependent calibration, not chosen; min. peak length, 2; max. charge, 5; min. score for recalibration, 70, cut peaks, chosen; gap scans, 1; advanced peak splitting, not chosen; intensity threshold, 500; intensity determination, value at maximum, label-free quantitation (LFQ) min. ratio count, 2; Fast LFQ, chosen; LFQ min. number of neighbors, 3; LFQ average number of neighbors, 6; number of modifications per peptide, 5; min. time, NaN; max. time NaN; additional var mods for special proteins, not chosen; separate variable modifications for first search, not chosen; separate enzyme for first search, not chosen.

Global parameters were chosen as follows: a fasta file containing all Uni-Prot listed *Arabidopsis thaliana* protein sequences; fixed modifications, carbamidomethyl (C); re-quantify, not chosen; match between runs, chosen; match time window, 0.7min; alignment time window, 20min; match unidentified features, not chosen; decoy mode, revert; special AAs, KR; include contaminants, chosen; I=L, not chosen; max peptide mass, 4600Da; min. peptide length for unspecific search, 8; max. peptide length for unspecific search, 25; PSM FDR, 0.01; protein fdr, 0.01; Site decoy fraction, 0.01; min. peptide length, 7; min. peptides, 1; min. razor + unique peptides, 1; min. unique peptides, 0; min. score for unmodified peptides, 0; min. score for modified peptides, 40; min. delta score for unmodified peptides, 0; min. delta score for modified peptides, 6; base FDR calculation of delta score, not chosen; razor protein FDR, chosen; split protein groups by taxonomy ID, not chosen; filter labelled amino acids, chosen; second peptides, chosen; dependent peptides, not chosen; min ratio count, 1.5; peptides for quantification, unique + razor; use only unmodified peptides and selected modifications, chosen; modifications used in protein quantification, acetyl (N-term), oxidation (M); discard unmodified counterpart peptide, chosen; separate LFQ in parameter groups, not chosen; stabilize large LFQ ratios, chosen; require MS/MS for LFQ comparisons, chosen; iBAQ, chosen; Log fit, chosen; advanced site intensities, chosen.

LFQ intensities from the corresponding MaxQuant ‘proteinGroups.txt’ file were uploaded into the Perseus software (http://www.biochem.mpg.de/5111810/perseus) to build a quantitation matrix. Data were cleaned from the matrix by applying the following parameters: columns identified only by site, reverse, potential contaminant; mode, remove matching rows; filter mode, reduce matrix. Categorical annotation of rows was performed manually (‘create’) and invalid data were removed by filtering rows based on valid values: min. number of values, 3; mode, in at least one group; values should be greater than 0; filter mode, reduce matrix. Two-sample testing was achieved by means of a *t*-test using the following parameters: S0; side, both; permutation-based FDR, 0.05, number of randomizations, 250; preserve grouping in randomizations, none; –log10, chosen. The –log10 *P*-value was calculated and the cut-off for the following analysis was *P*-value >1.31. Localization of proteins was analysed with SUBAcon (Tanz *et al.*, 2013; Hooper *et al.*, 2014) and the functional context with MapMan (Thimm *et al.*, 2004).

### Oxygen consumption measurements

Oxygen consumption of isolated mitochondria was measured using a Clark-type oxygen electrode (Hansatech Instruments, Norfolk, UK) according to Meyer *et al.* (2009). The reaction buffer included 100 µg mitochondrial protein in 3ml respiration buffer (300mM sucrose, 5mM KH_2_PO_4_, 10mM TES, 10mM NaCl, 2mM MgSO_4_, 0.1% [w/v] BSA, pH 7.2) supplied with 5mM succinate and 500 µM ATP. At stable oxygen consumption, 200 µM ADP was added for measuring ADP-dependent respiration. For estimation of AOX capacity, 500 µM of AOX inhibitor n-propyl gallate (nPG) was added and the O_2_ consumption rate after adding nPG was subtracted from the ADP-dependent O_2_ consumption rate.

## Results

### Comparison of the mitochondrial proteomes of wt and *ca1ca2* lines using 2D IEF/SDS PAGE

To investigate the consequences of the absence of complex I on the mitochondrial compartment, comparative proteome analyses of wt and *ca1ca2* mitochondria isolated from cell culture lines were performed. Proteins were separated by 2D IEF/SDS PAGE and spot volumes were systematically compared using the Delta 2D software package (Decodon, Greifswald).

**Fig. 1.**
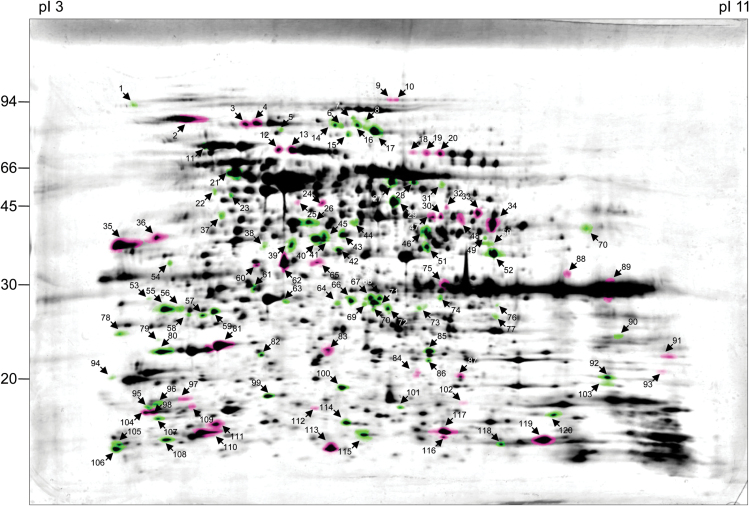
Comparative analysis of the mitochondrial proteomes of Arabidopsis wt and *ca1ca2* lines. Mitochondria were isolated as described in the Materials and Methods. Total mitochondrial protein was separated by 2D IEF/SDS PAGE and proteins were stained by Coomassie blue. Three replicates were produced per line and used for the calculation of master gels (Delta 2D software package, Decodon, Germany). The molecular masses of standard proteins are given to the left of the 2-D gel (in kDa). Isoelectric focusing range is from pH 3 (left) to pH 11 (right). Proteins indicated in pink are more abundant in the mutant (>1.5-fold increase); proteins indicated in green are less abundant in the mutant (>1.5-fold decrease). Spots indicated by numbers were identified by mass spectrometry (for results see Supplementary Table S1).

Volumes of 121 spots were significantly altered, with a fold change of >1.5 (*P*-value < 0.01) between wt and *ca1ca2* lines. Forty-four spots had higher volume in the *ca1ca2* mutant, whereas 77 spot volumes were increased in wt (reduced in *ca1ca2*) (Fig. 1). All 121 spots were analysed by mass spectrometry. After applying a MASCOT threshold score of 60, overall a total of 288 identified proteins were included in further analyses. More than one protein was identified for several spots. Changes in volume were only assigned to a specific protein if a spot only included one main protein. This further reduced the number of unambiguously changed proteins to 106. Sixty-six of these proteins were of decreased abundance in the mutant and 40 of increased abundance. These proteins were annotated according to their functional context (Supplemantary Table S1 at *JXB* online).

As expected, many proteins of reduced abundance were complex I subunits (15 proteins). We did not find all the complex I proteins because 2D IEF/SDS PAGE does not allow separation of very hydrophobic proteins. Reduction of complex I subunits was on average 5-fold. It was shown previously by Fromm *et al.* (2016*c*) that complex I is completely absent in the mutant (Supplemantary Fig. S1); however, non-assembled subunits may be present in mitochondria. Furthermore, reduction of levels of complex I subunits can be expected to be even higher because nearly all spots included not only one main protein but also in addition some proteins of low abundance, which probably were of unchanged or not much changed abundance. As a consequence, fold-changes in general might be slightly higher than determined in the frame of our study. Other proteins of reduced abundance in the mutant are involved in the central mitochondrial metabolism whereas many proteins of increased abundance play roles in transport or stress response processes (Table 1).

**Table 1.** Relative spot volumes of altered proteins involved in defined functional processes in ca1ca2 mutant lines. Proteins were separated by 2D IEF/SDS PAGE (Fig. 1), normalized spot volumes of differential OXPHOS subunits were summed up and relative spot volumes were calculated by the Delta 2D software package

Functional context (number of proteins of changed abundance)	Relative spot volume with respect to wt plants (%)
Stress response (12)	186
Transport (7)	159
Processing of nucleic acids (5)	157
Protein folding and processing (6)	143
Oxidative phosphorylation without complex I (18)	133
Uncharacterized (4)	111
Miscellaneous proteins (6)	102
Amino acid metabolism (7)	75
Lipid metabolism (9)	68
TCA cycle (5)	45
Carbon fixation (4)	28

Detailed evaluation of the dataset revealed the following. Changes in abundance of TCA cycle enzymes were not uniform. We found subunits of citrate synthase (AT2G44350), malate dehydrogenase (AT3G15020), and succinyl-CoA ligase (AT5G08300) of decreased abundance in the *ca1ca2* mutant, whereas a subunit of the oxoglutarate dehydrogenase complex (AT5G55070) was increased. Glutamate dehydrogenase (AT5G18170) was also increased in the mutant. Notably, several subunits of the TIM translocase were increased (TIM8, TIM9, TIM23). Several of the most induced proteins in the mutant are involved in plant stress responses.

Some of the proteins of changed abundance were subunits of the remaining OXPHOS complexes II–V. Again, not all of the subunits were identified because many of them are very hydrophobic and not resolvable by 2D IEF/SDS PAGE. It became apparent that several complex IV subunits are clearly induced. To get a more complete impression on how the mutant is altered with respect to the OXPHOS system, we next compared mitochondrial fractions of wt and *ca1ca2* lines using 2D BN/SDS PAGE, a gel electrophoresis system known to be particularly suitable for analysing membrane-bound proteins and protein complexes.

### Comparison of the mitochondrial proteomes of wt and *ca1ca2* lines using 2D BN/SDS PAGE

As previously reported, the *ca1ca2* mutant shows changes in the activities of complex II and complex IV (Fromm *et al.*, 2016*c*). In order to evaluate changes in the amounts of the OXPHOS complexes II–V in the absence of complex I in more detail, comparative proteome experiments by 2D BN/SDS PAGE were performed with mitochondrial membrane fractions of wt and *ca1ca2* cell culture lines. The comparisons were based on two methods: (i) Delta 2D-mediated comparison of 2-D gels, and (ii) fluorophore based comparison (2D DIGE).

**Fig. 2.**
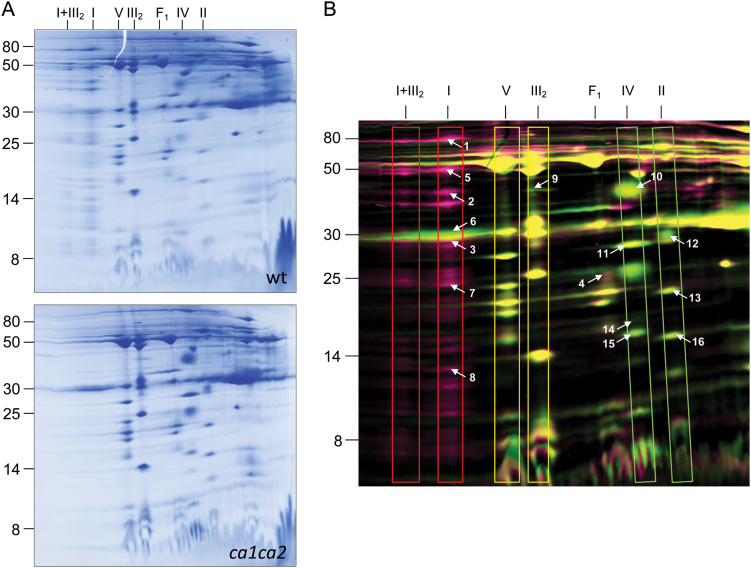
Comparative analysis of the mitochondrial membrane proteomes of Arabidopsis wt and *ca1ca2* lines. Mitochondria were isolated as described in the Materials and methods. Mitochondrial membrane proteins were separated by 2D BN/SDS PAGE and proteins were stained by colloidal Coomassie (A). Three replicates were produced per fraction and used for the calculation of a master gel (Delta 2D software package, Decodon, Germany) (B). The molecular masses of standard proteins are given to the left of the 2-D gels (in kDa). OXPHOS complexes are boxed in (B); their identities are given above the gels (I, complex I; V, complex V; III_2_, dimeric complex III; I+III_2_, supercomplex formed of complex I and dimeric complex III; F_1_, F_1_ part of complex V; IV, complex IV; II, complex II). Proteins indicated in pink are less abundant in the mutant (>1.5-fold decrease); proteins indicated in green are more abundant in the mutant (>1.5-fold increase). Spots indicated by numbers were identified by mass spectrometry (for results see Supplementary Table S2).

Visual inspection of the 2D BN/SDS gels used for Delta 2D comparison clearly revealed the absence of complex I and the I+III_2_ supercomplex in the mutant (Fig. 2A). On the resulting overlay image (Fig. 2B) the complexes III_2_ and V are more-or-less unchanged, while the complexes II and IV are clearly increased in the mutant. Average spot volumes were calculated for each complex in the two fractions using the Delta 2D software (Fig. 2B, Table 2). The following amounts of the OXPHOS complexes were found for the mutant (wt=100%): complex II, 133%; complex III, 108%; complex IV, 200%; and complex V, 107%. Proteins within 16 spots were analysed by MS (Supplemantary Table S2) and all revealed the expected identifications (see the 2D BN/SDS GelMap of the Arabidopsis mitochondrial proteome for comparison, https://gelmap.de/1227).

**Table 2.** Relative spot volumes of OXPHOS complexes in ca1ca2 lines. Proteins were separated by 2D BN/SDS PAGE (Fig. 2), normalized spot volumes of differential OXPHOS subunits were summed up and relative spot volumes were calculated by the Delta 2D software package

OXPHOS complex	relative spot volume with respect to wt plants (%)
Complex I	- *
Complex II	133
Complex III	108
Complex IV	200
Complex V	107

* not detectable

**Fig. 3.**
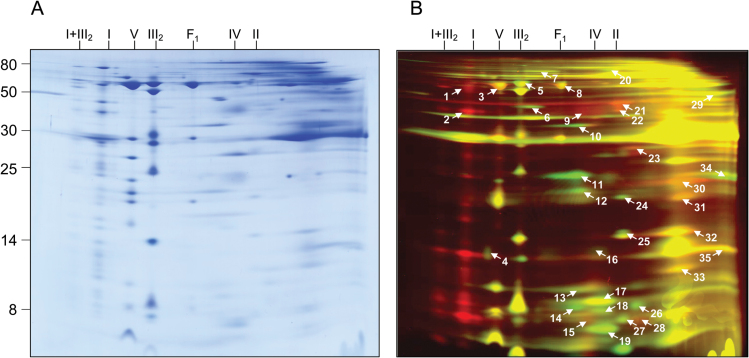
Comparative analysis of the mitochondrial membrane proteomes of Arabidopsis wt and *ca1ca2* lines by differential gel electrophoresis (DIGE). Mitochondria were isolated as described in the Materials and Methods. Mitochondrial membrane proteins of wt and *ca1ca2* were labeled with different CyDyes and separated by 2D BN/SDS PAGE. Proteins were stained by colloidal Coomassie (A). The same gel was used for fluorescence detection of the two CyDyes (B). The molecular masses of standard proteins are given to the left of the 2-D gel (in kDa). The identities of selected mitochondrial protein complexes are given above the gels (I, complex I; V, complex V; III_2_, dimeric complex III; I+III_2_, supercomplex formed of complex I and dimeric complex III; F_1_, F_1_ part of complex V; IV, complex IV; II, complex II). Proteins indicated in red are less abundant in the *ca1ca2* mutant (>1.5-fold decrease) and proteins indicated in green are more abundant in the *ca1ca2* mutant (>1.5-fold increase). Proteins given in yellow are not changed in abundance. Spots indicated by numbers were identified by mass spectrometry (for results see Supplementary Table S3). Note: if compared to the comparative experiment shown in Fig. 2, several subunits of OXPHOS complexes appear to be absent in the DIGE approach. This is due to the fact that CyDye labeling takes place at native conditions. As a consequence, only proteins exposed to the surface of protein complexes are labeled. In contrast, image evaluation based on the Delta 2D approach (Fig. 2) allows visualization of all subunits of a protein complex.

A more extended comparison of the mitochondrial membrane proteomes of mutant and wt cell lines was carried out based on 2D BN/SDS DIGE (Fig. 3, Supplemantary Table S3). Spots differing in volumes between the two fractions were analysed by MS. After applying a MASCOT threshold score of 60, overall 147 identified proteins were included in further analyses; however, a difference in spot volume only could be assigned to a specific protein if a spot included only one main protein. This further reduced the number of unambiguously changed proteins to 44. These were grouped according to functional context. Changes of individual subunits of OXPHOS complexes were in accordance with the results of the Delta 2D analysis. In addition, several other membrane proteins were found to be of changed abundance in the mutant. Seven subunits of the TIM and TOM transport machineries and one ABC transporter were identified. All were more abundant in the *ca1ca2* mutant.

### Comparison of total protein extracts of wt and *ca1ca2* lines by label-free quantitative shotgun proteomics

In addition to effects on the mitochondrial compartment, the consequences of *ca1ca2* deletion at the whole-plant level were investigated. Wild type and *ca1ca2* mutant plants were harvested at a comparable growth stage and differential protein abundances were analysed by comparative quantitative shotgun MS (note that growth and development of *ca1ca2* plants is much delayed; see Fromm *et al.*, 2016c). The experiment was based on five biological replicates. In total, 2233 different proteins were identified. The quantitative analysis of identified proteins was carried out using MaxQuant. After application of a –log10 *P*-value (*P*>1.31), 318 proteins of changed amounts were confirmed (Supplemantary Table S4) and were included in further analyses.

**Fig. 4.**
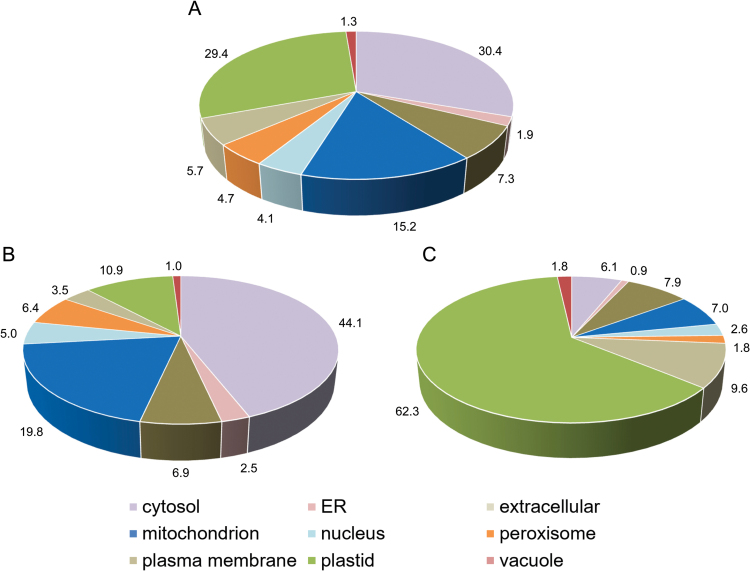
Subcellular localization of proteins of altered abundances in the *ca1ca2* line as obtained by label free quantitative shotgun proteomics. Total protein was extracted from wt and *ca1ca2* mutant plants at a similar developmental stage. Proteins were identified and quantified by shotgun MS (for details see the Material and Methods). Predicted localizations of proteins of changed abundances between the two lines were obtained from the SUBA3 database (http://suba3.plantenergy.uwa.edu.au/). (A) Predicted localization of all proteins changed (*P*-value <0.05). (B) Predicted localization of proteins more abundant in *ca1ca2* compared to wt. (C) Predicted localization of proteins less abundant in *ca1ca2* mutant compared to wt. Numbers indicate amounts relative to the sum of altered protein species (%).

The proteins were assigned into categories according to subcellular localization and functional context. Subcellular localization was assigned according to SUBAcon (Fig. 4). Most of the identified proteins are localized in the cytosol (30.4%), followed by plastids (29.4%), mitochondria (15.2%), and other compartments with minor contributions (Fig. 4A). Proteins of increased abundance in *ca1ca2* plants are mostly localized in the cytosol (44.1%) and mitochondria (19.8%) (Fig. 4B). In contrast, proteins of decreased abundance in *ca1ca2* plants are mostly localized in plastids (62.3%) (Fig. 4C).

**Fig. 5.**
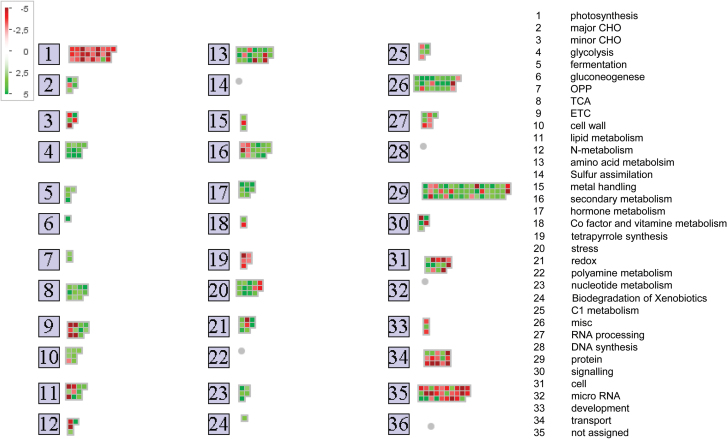
Functional annotation of proteins identified by quantitative label-free shotgun MS. The 318 proteins of differential abundances between wt and *ca1ca2* mutant plants were grouped into functional BINs using MapMan. BINs are given to the right. Proteins more abundant in *ca1ca2* are indicated in green and proteins that are less abundant in red (*P*-value <0.05). Each square represents one protein. BINs without any identified protein are with a grey dot (for results see Supplementary Table S4).

Assignment of proteins differing in amount between wt and *ca1ca2* mutant plants to functional categories was carried out according to TAIR functional annotations (https://www.arabidopsis.org/, TAIR10 genome release) and evaluated by MapMan (Fig. 5, Table 3). Proteins especially induced in mutant plants are involved in glycolysis, fermentation, the TCA cycle, amino acid metabolism, redox regulation, protein folding, as well as stress responses (Supplemantary Table S4). Decreased protein abundances in *ca1ca2* plants were mainly found in the functional categories of photosynthesis (photosystem I, photosystem II, Calvin cycle, photorespiration) and tetrapyrrole synthesis (Fig. 5). As expected, complex I subunits were much decreased in the mutant. At the same time, AOX1A (AT3G22370) and alternative NADH dehydrogenase NDB2 (AT4G05020) were clearly induced.

**Table 3.** Relative protein intensities of altered proteins involved in defined functional processes in ca1ca2 mutant lines analysed by label-free quantitative shotgun MS approach. Proteins were identified and quantified using MaxQuant software

Functional context (number of proteins of changed abundance)	Relative protein intensities with respect to wt plants (%)
Fermentation (4)	183
Glycolysis (10)	173
Protein folding (9)	172
Oxidative pentose phosphate (2)	166
Signalling (5)	162
Redox (8)	160
TCA cycle (11)	159
Stress response (14)	152
Amino acid metabolism (17)	149
Mitochondrial electron transport without complex I (5)	144
Cell wall (7)	140
Cell organisation (14)	132
Miscellaneous proteins (25)	121
Lipid metabolism (10)	120
Secondary metabolism (13)	114
Protein processing (6)	110
Protein degradation (18)	102
Protein synthesis (11)	95
Co-factor and vitamine metabolism (2)	89
Development (3)	89
Metal handling (2)	89
Protein targeting (7)	85
Uncharacterized (29)	81
Carbon metabolism (14)	79
Transport (15)	78
Photorespiration (4)	75
Calvin cycle (9)	66
Photosystem II (4)	66
Photosystem I (2)	66
Tetrapyrrole synthesis (5)	65
Light reaction others (3)	64
N-metabolism (4)	64
Processing of nucleic acids (11)	63
ATP synthase (plastid) (3)	61

Finally, we analysed the BIN coverage of the identified proteins in order to assess the influence of the absence of complex I on cellular processes (Table 4). We calculated the number of identified proteins in relation to the number of genes that code for proteins of the BINs. The following BINs were most significantly changed in the mutant: fermentation (28.6%), glycolysis (15.4%), nitrogen metabolism (15.4%), TCA cycle (13.8%), photosynthesis (12.6%), tetrapyrrole synthesis (11.1%), mitochondrial ETC (7.5%), and amino acid metabolism (6.8%). The BIN coverage indicates that these cellular process are particularly affected by absence of complex I.

**Table 4.** BIN coverage of proteins of altered abundances in ca1ca2 plants as determined by label-free quantitative shotgun MS

MapMan BIN	BIN name	Sum of genes	Number of differential proteins in wt and *ca1ca2*	Number of differential proteins / sum of genes per BIN (%)
5	Fermentation	14	4	28.6
4	Glycolysis	65	10	15.4
12	N-metabolism	26	4	15.4
8	TCA	80	11	13.8
1	Photosynthesis	199	25	12.6
19	Tetrapyrrole synthesis	45	5	11.1
25	C1 metabolism	39	4	10.3
6	Gluconeogenese	10	1	10.0
9	Mitochondrial electron transport	146	11	7.5
13	Amino acid metabolism	251	17	6.8
7	Oxidative pentose phosphate pathway	31	2	6.5
2	Major carbon metabolism	100	5	5.0
21	Redox	194	8	4.1
3	Minor carbon metabolism	124	5	4.0
24	Biodegradation of Xenobiotics	27	1	3.7
15	Metal handling	67	2	3.0
16	Secondary metabolism	438	13	3.0
23	Nucleotide metabolism	169	5	3.0
18	Co-factor and vitamine metabolism	79	2	2.5
11	Lipid metabolism	398	10	2.5
31	Cell organisation	746	14	1.9
26	Miscellaneous proteins	1397	25	1.8
20	Stress response	874	14	1.6
34	Transport	996	15	1.5
29	Protein folding and processing	3409	51	1.5
10	Cell wall	496	7	1.4
17	Hormone metabolism	495	5	1.0
33	Development	681	3	0.4
30	Signalling	1239	5	0.4
35	Not assigned	7748	29	0.4
27	RNA processing	2567	6	0.2
14	Sulfur assimilation	12	0	0.0
32	Micro RNA	4	0	0.0
28	DNA synthesis	1352	0	0.0
22	Polyamine metabolism	18	0	0.0

**Fig. 6.**
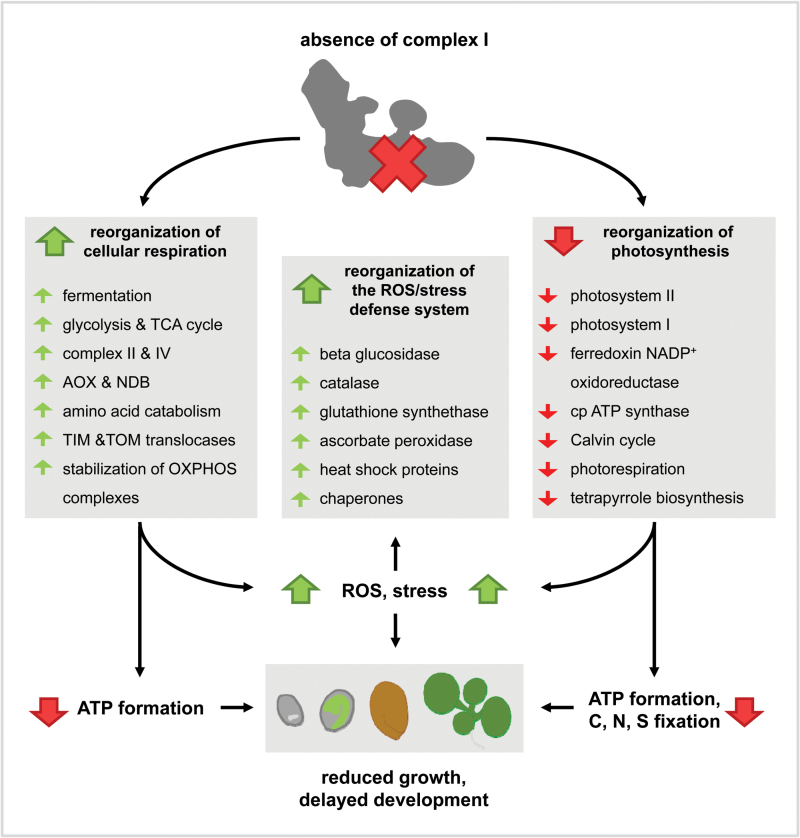
Life without complex I. The figure is based on altered protein levels in *ca1ca2* plants relative to wildtype plants as obtained by label-free quantitative shotgun proteomics. Green arrows within the grey boxes indicate increased protein levels in the double-mutant, and red arrows indicate decreased levels. Absence of complex I causes reorganization of the cellular respiration system. Since electron insertion into the first segment of the mETC is not possible, increased electron insertion at later segments takes place (induction of complexes II, IV). This requires increased oxidation of organic substrates (induction of enzymes of glycolysis, the TCA cycle, and amino acid catabolism). Mitochondrial ATP formation most likely is still reduced, which requires increased fermentation. The growth rate of the double-mutant is drastically reduced. This is reflected by reduced amounts of the two photosystems, Calvin cycle enzymes, and enzymes of the tetrapyrrole biosynthesis pathway. Furthermore, altered metabolism and electron transport pathways in the mitochondria and chloroplasts cause increased ROS formation and stress symptoms. Several components of the ROS and stress defense system are induced in the double-mutant, as is the alternative oxidase, a well-known stress indicator in plants. Note: causal events indicated by black arrows do not necessarily indicate primary effects, but may well represent indirect consequences. For further details see the discussion section.

Changes in protein levels in *ca1ca2* plants with respect to wildtype plants as obtained by label-free quantitative shotgun proteomics are summarized in Fig. 6 and in the discussion below.

## Discussion

In previous studies, the *ca1ca2* mutant has been characterized with respect to development, mitochondrial metabolism, and features of the OXPHOS system (Fromm *et al.*, 2016c). In order to systematically monitor the consequences of the double gene deletion on the mitochondrial proteome and the entire leaf proteome, we report here the results of three different experimental approaches, two of which were based on gel electrophoresis and one on gel-free shotgun proteomics. All three experimental systems have advantages and limitations. 2D IEF/SDS PAGE-based analyses excel in investigating prominent hydrophilic proteins. 2D BN/SDS PAGE is strong in analysing membrane proteins and membrane-bound protein complexes, which also are relatively abundant. Shotgun proteome analysis is a less systematic approach that allows the analysis of a very large number of proteins at the same time, and as such it better covers proteins of comparatively low abundance than the two gel-based approaches. Furthermore it should be noted that the 2D IEF/SDS and 2D BN/SDS PAGE approaches were carried out using cell cultures whereas shotgun proteomics was performed on total protein extracts from leaves. Cell suspension cultures are always well supplied with sucrose, whereas plants have to generate sugars by photosynthesis on their own. Therefore, the outcomes of the three experimental approaches need to be compared critically (Tables 1 and 3).

Although the three experimental systems and the sources of the analysed protein fractions differed, all the approaches gave similar results in respect to proteomic alterations taking place in the *ca1ca2* double-knock-out mutant. As expected, all the approaches indicated a dramatic reduction of complex I subunits. However, residual levels of non-assembled complex I proteins seem to be present in the mutant’s mitochondria.

Levels of complex II and especially complex IV were much higher in the double-mutant, as were the levels of some of the alternative oxidoreductases of the plant mitochondrial OXPHOS system. This has been previously reported for other complex I mutants (Keren *et al.*, 2012; Hsu *et al.*, 2014). Upregulation of complex IV points to elevated proton translocation at the final segment of the mETC, which could partially compensate for the diminished proton translocation at the first segment of the mETC in the absence of complex I. Indeed, increased *in vitro* activity of complex IV in the *ca1ca2* mutant has been shown previously (Fromm *et al.*, 2016c). An increased oxygen consumption of *ca1ca2* mitochondria was observed if succinate was used as substrate (Supplemantary Fig. S2). This might indicate a higher complex II activity, but could also be the consequence of an elevated electron flux through the mETC caused by higher complex IV activity.

In contrast, levels of the complexes III and V were rather similar in the *ca1ca2* and wt lines, although relative abundances of some subunits of these complexes were altered (Supplemantary Tables S1 and S2). The overall upregulation of the OXPHOS complexes in mutant cells requires increased import rates of the corresponding nuclear encoded subunits by the TOM and TIM pre-protein import machineries (Murcha *et al.*, 2014). Indeed, levels of TIM and TOM subunits clearly went up in the double-mutant (Fig. 1, Supplemantary Table S3).

The alternative NADH dehydrogenase NDB2 (AT4G05020) and the alternative oxidase AOX1A (AT3G22370) were induced 4- and 8-fold higher in the mutant (Supplemantary Table S4). This corresponds to increased capacity for alternative oxidase in the *ca1ca2* mutants (Supplemantary Fig. S2). An increase of AOX has been previously reported for several plant complex I mutants, e.g. the *nmat1*, *opt43*, *cmsII*, *ndufv1*, and *mTerf15* lines (Gutierres *et al.*, 1999; de Longevialle *et al.*, 2007; Keren *et al.*, 2012; Hsu *et al.*, 2014; Kühn *et al.*, 2015; see Table 5 for information on complex I mutants discussed in this section). Indeed, increased oxygen consumption rates have been observed for some complex I mutants, e.g. the *ca1ca2* and *ndufv1* lines (Kühn *et al.*, 2015; Fromm *et al.*, 2016c).

**Table 5.** Summary of complex I mutants in plants

Name of mutant	Mutation of ...	Complex I depletion (i) or absence of complex I (ii)	Plant species	Reference
*ca2*	Complex I subunit	i	*Arabidopsis thaliana*	Perales *et al.*, 2005 & Fromm *et al.*, 2016c
*ca1ca2*	Complex I subunit	ii	*Arabidopsis thaliana*	Fromm *et al.*, 2016c
*cal1cal2i*	Complex I subunits	i	*Arabidopsis thaliana*	Fromm *et al.*, 2016b
*ca2cal1 or ca2cal2*	Complex I subunits	i	*Arabidopsis thaliana*	Soto *et al.*, 2015
*ndufs4*	Complex I subunit	i	*Arabidopsis thaliana*	Kühn *et al.*, 2015
*ndufv1*	Complex I subunit	ii	*Arabidopsis thaliana*	Kühn *et al.*, 2015
*gldh*	Assembly factor	ii	*Arabidopsis thaliana*	Pineau *et al.*, 2008
*indh*	Assembly factor	ii	*Arabidopsis thaliana*	Wydro *et al.*, 2013
*opt43*	*NAD1* splicing factor	ii	*Arabidopsis thaliana*	de Longevialle *et al.*, 2007
*nMat1*	*NAD1* splicing factor	ii	*Arabidopsis thaliana*	Keren *et al.*, 2012
*mTERF15*	*NAD2* splicing factor	ii	*Arabidopsis thaliana*	Hsu *et al.*, 2014
*nms1*	*NAD4* splicing factor	i	*Nicotiana sylvestris*	Brangeon *et al.*, 2000
*slo3*	*NAD7* splicing factor	i	*Arabidopsis thaliana*	Hsieh *et al.*, 2015
*bir6*	*NAD7* splicing factor	i	*Arabidopsis thaliana*	Koprivova *et al.*, 2010
*cmsII*	Leads to NAD7 deletion	ii	*Nicotiana sylvestris*	Gutierres *et al.*, 1999
*ncs2*	Replacing the 3′-end of *NAD4* with sequences from *NAD7*	ii	*Zea mays*	Karpova *et al.*, 2002

Induction of the alternative oxidoreductases of the respiratory chain is known to be an integral part of the general plant stress response (Vanlerberghe, 2013). This, together with the induction of a large number of further stress related proteins [such as beta glucosidases (AT1G66270, AT3G09260, AT3G09260, AT3G16420), catalase 3 (AT1G20620), ascorbate peroxidase 4 (AT4G09010) and glutathione syntethase 2 (AT5G27380)] in the double-mutant strongly indicates that the absence of complex I strongly affects the metabolic balance and the redox homeostasis of the plant cell.

High levels of complex II, complex IV, and alternative oxidoreductases of the mETC require increased provision of electrons to the mETC. Indeed, the enzymes involved in glycolysis were clearly induced in the double-mutants. It has been shown previously for the *ndufv1* mutant, which also completely lacks mitochondrial complex I, that complex I deficiency causes a metabolic switch and that flux through glycolysis significantly increases (Kühn *et al.*, 2015). Furthermore, our data point to an extended usage of fermentation to compensate for decreased ATP generation and decreased capacity of NADH oxidation by the complex I-deficient mETC. In contrast, the proteomic changes with respect to the citric acid cycle are more difficult to understand, as some enzymes are reduced whereas others are increased in the *ca1ca2* double-mutant. This points to a scenario that the absence of complex I does not induce the entire citric acid cycle, but rather specific segments of the pathway. Indeed, it is known that plant mitochondria have, depending on the physiological state of the respective cell, quite a number of non-cyclic operation modi with respect to the citric acid cycle (Sweetlove *et al.*, 2010).

Electrons for the mETC can also originate from amino acid breakdown (Sweetlove *et al.*, 2010; Schertl and Braun 2014; Hildebrandt *et al.*, 2015). Several proteins involved in mitochondrial amino acid catabolism were identified by our shotgun proteome approach and found to be induced in the *ca1ca2* mutant, e.g. alanine, tyrosine, and branched-chain aminotransferases (AT1G17290, AT4G23600, AT3G19710), glutamate dehydrogenase (AT5G18170, AT5G07440), arginase (AT4G08870), and methylmalonate-semialdehyde dehydrogenase (AT2G14170). The latter enzyme is involved in a step of branched-chain amino acid oxidation. The number of electrons provided for the mETC from amino acid breakdown is especially high during oxidation of the branched chain amino acids (Hildebrandt *et al.*, 2015).

Prohibitins and stomatin-like proteins (SLP) were very much increased in the *ca1ca2* mutant, as revealed by all three proteome analyses. In animal cells respiratory supercomplexes are stabilized by cardiolipin and SLPs. SLPs can bind cardiolipin and interact with prohibitins (Mitsopoulos *et al.*, 2015). Similar interactions have also been reported for the mitochondria of plants (Gehl *et al.*, 2014; Gehl and Sweetlove, 2014). Knock-out mutants for *slp1* have reduced complex I levels and activity, and form lower amounts of supercomplexes, indicating that SLPs and prohibitins can affect the assembly and/or the stability of OXPHOS complexes (Gehl *et al.*, 2014). Complex I subunits that cannot be assembled might be stabilized to a certain degree by prohibitins and SLPs. Furthermore, prohibitins play a role in mitochondrial DNA organization, stress tolerance, and triggering retrograde signals in response to stress and mitochondrial dysfunction (Van Aken *et al.*, 2010).

The phenotype of complex I mutant plants often includes curled leaves and a delayed vegetative and reproductive development (de Longevialle *et al.*, 2007; Meyer *et al.*, 2009; Wang *et al.*, 2012; Kühn *et al.*, 2015; Hsu *et al.*, 2014). The degree of the developmental delay and the curly leaf phenotype are dependent on the amount of residual complex I (Kühn *et al.*, 2015). For example, trace amounts of complex I are sufficient for plants to pass through embryogenesis, whereas mutants lacking complex I, such as *cal1cal2*, *opt43*, *indh*, *ndufv1*, and *ca1ca2*, cannot complete this developmental stage and hardly germinate (de Longevialle *et al.*, 2007; Wang *et al.*, 2012; Wydro *et al.*, 2013; Kühn *et al.*, 2015; Fromm *et al.*, 2016c). The growth rate of the *ca1ca2* mutant has been reported to be drastically reduced (Fromm *et al.*, 2016c). This is clearly reflected by our shotgun proteome data.

Overall, proteins involved in developmental processes and photosynthesis were very much reduced in the *ca1ca2* mutant. More than 60% of the proteins of lower abundance in the *ca1ca2* mutant are localized in plastids (Fig. 4C). All detected subunits of the two photosystems (PS) were reduced, as were the ferredoxin-NADP^+^ oxidoreductase (AT5G66190, AT1G20020) and the subunits of the chloroplast ATP synthase complex. The maize *ncsII* and *ncs6* mutants also have a decrease in PSI while other photosynthetic complexes are unaffected. The chloroplast ultrastructure is abnormal (Roussel *et al.*, 1991; Jiao *et al.*, 2005). Two recently described small twin cystein proteins, which are specifically induced in complex I-deficient plants, also specifically affect chloroplast metabolism (Wang *et al.*, 2016). Furthermore, enzymes involved in the Calvin cycle are of reduced abundance in *ca1ca2* plants. This indicates substantial consequences of the absence of complex I on photosynthesis. Additionally, tetrapyrrole synthesis is impaired. Tetrapyrroles are essential for chlorophyll biosynthesis. It has been suggested that reduction of photosynthetic proteins may be caused by impaired chlorophyll synthesis (Brzezowski *et al.*, 2015). Our results are in line with those obtained for the *cmsII* mutant of *Nicotiana sylvestris*, which also lacks complex I. In *cmsII* mutants photosynthetic efficiency is reduced (Sabar *et al.*, 2000; Dutilleul *et al.*, 2003). Defects in the photosystems may result in ROS formation (Schmitt *et al.*, 2014). Higher ROS levels have indeed been observed in the *ca1ca2* and other complex I mutants (Keren *et al.*, 2012; Córdoba *et al.*, 2016; Fromm *et al.*, 2016c).

ROS may cause cellular damage and programmed cell death (Li and Xing, 2010), and thus they negatively affect plant development. Our proteome data indicate that seed photosynthesis in *ca1ca2* mutant embryos may also be impaired by complex I dysfunction. A higher ROS content has been found in *ca1ca2* embryos (Córdoba *et al.*, 2016, Ostersetzer-Brian 2016). Defects in the photosynthetic apparatus should cause decreased synthesis and accumulation of seed storage compounds, which will be further impaired by mitochondrial dysfunction (Schwender *et al.*, 2006). Seed storage compounds such as fatty acids are essential to drive the germination process (Carrie *et al.*, 2013). *ca1ca2* mutant embryos depleted in energy-rich components are not able to develop normally during germination, which results in seed abortion (Córdoba *et al.*, 2016, Fromm *et al.*, 2016c).

Reduced photosynthesis affects photorespiration. Indeed, all the identified proteins of the photorespiration pathway were reduced in the *ca1ca2* mutant. This also applies for the T and the P subunits of the mitochondrial glycine decarboxylase complex (GDC) (AT1G11860; AT2G26080). Down-regulation of the GDC complex has been reported to be caused either by impaired photosynthesis or by feedback inhibition by an elevated NADH pool in the matrix (Oliver, 1994; Peterhänsel *et al.*, 2010), which may be caused by the absence of complex I.

Besides the absence of the electron transfer function of complex I, which is coupled to proton translocation across the inner mitochondrial membrane, complex I is assumed to include further enzymatic and transport functions (Braun *et al.*, 2014). In particular, the complex I-integrated carbonic anhydrase subunits have been suggested to play a role in recycling mitochondrial CO_2_ for carbon fixation in the chloroplasts (Zabaleta *et al.*, 2012). Thus it may well be that the *ca1ca2* mutant lacks more than just the NADH-ubiquinone-oxidoreductase activity. However, the *ca1ca2* mutant very much behaves like other mutants that completely lack complex I, e.g. *ndufv1* (Kühn *et al.*, 2015). Furthermore, in mutants lacking complex I due to the absence of other complex I subunits the formation of the carbonic anhydrase domain is also prevented. This makes the *ca1ca2* mutant an excellent model for studying the role of mitochondrial complex I in plants, the proteomic level of which has been addressed in this study.

## Conclusions

‘Life without complex I’ is not so easy, even in plants that possess some alternative dehydrogenases in the mitochondrial compartment (which, however, do not contribute to the proton gradient across the inner mitochondrial membrane). The metabolic balance of the plant cell is deeply disturbed in the absence of complex I. Complex I dysfunction causes reorganization of cellular respiration and affects metabolic processes in mitochondria, plastids, peroxisomes, and other cellular compartments with drastic consequences for growth and development. Specifically, proteins involved in glycolysis and the TCA cycle are induced, as are subunits of other OXPHOS complexes, especially the complex IV. This requires an upregulation of the TIM and TOM translocases for mitochondrial protein import. Furthermore, alternate electron entry pathways into the mETC are induced, e.g. oxidation of amino acids. Increased flux of electrons through the mETC causes elevated ROS formation in *ca1ca2* plants. ATP formation in the chloroplasts is reduced by decreased photosynthesis, e.g. caused by defective chlorophyll biosynthesis in *ca1ca2* mutant plants. In summary, plant cells metabolically rearrange in the absence of complex I in order to maintain a minimum level of energy supply and to balance redox homeostasis. At the same time, *ca1ca2* mutants suffer from increased ROS production and reduced ATP generation by both, respiration and photosynthesis.

## Supplementary data

Supplementary data are available at *JXB* online.

Figure S1. Growth phenotype of *ca1ca2* plants, in the absence of complex I.

Figure S2. Respiration through complex II and the AOX capacity of mitochondria derived from *Arabidopsis thaliana* wildtype (wt) and *ca1ca2* double-mutant lines.

Table S1. (a) Proteins of altered abundance in the *ca1ca2* mutant as revealed by 2D IEF/SDS PAGE (see Fig. 1). (b) Short version of (a).

Table S2. Identification of proteins from wt and *ca1ca2* mutant lines after separation by 2D BN/SDS PAGE and gel evaluation by Delta 2D software (see Fig. 2).

Table S3. Identification of proteins from wt and *ca1ca2* mutant lines after analysis by 2D BN/SDS DIGE (see Fig. 3).

Table S4. (a) Proteins of altered abundance in the *ca1ca2* mutant as obtained by label-free quantitative shotgun MS analysis. (b) Short version of (a).

Supplementary Data

## Abbreviations:

2D BN/SDS PAGE: two-dimensional blue native/sodium dodecyl sulfate polyacrylamide gel electrophorese
2D IEF/SDS PAGE: two-dimensional isoelectric focusing/sodium dodecyl sulfate PAGE
CA: carbonic anhydrase
CAL: carbonic anhydrase-like
complex I: NADH dehydrogenase complex
DIGE: difference gel electrophorese
OXPHOS: oxidative phosphorylation
ROS: reactive oxygen species
TCA cycle: tricarboxylic acid cycle
TIM: translocase of inner mitochondrial membrane
TOM: translocase of outer mitochondrial membrane.
