# Supplementary material for: Life without complex I: proteome analyses of an Arabidopsis mutant lacking the mitochondrial NADH dehydrogenase complex
Source: J Exp Bot. 2016 Apr 27;67(10):3079–93. doi: 10.1093/jxb/erw165 (PMC4867900; doi:10.1093/jxb/erw165)
Supplement: Supplementary Data [file supp_67_10_3079__index.html]

Life without complex I: proteome analyses of an Arabidopsis mutant lacking the mitochondrial NADH dehydrogenase complex — Life without complex I: proteome analyses of an Arabidopsis mutant lacking the mitochondrial NADH dehydrogenase complex — Supplementary Data 

# Life without complex I: proteome analyses of an Arabidopsis mutant lacking the mitochondrial NADH dehydrogenase complex

## Supplementary Data

Data files

- supplementary\_figures\_S1\_S2.pdf - Supplementary Data
- supplementary\_tables\_S1\_S4.xlsx - Supplementary Data
