# Supplementary material for: Life without complex I: proteome analyses of an Arabidopsis mutant lacking the mitochondrial NADH dehydrogenase complex
Source: J Exp Bot. 2016 Apr 27;67(10):3079–93. doi: 10.1093/jxb/erw165 (PMC4867900; doi:10.1093/jxb/erw165)
Supplement: Supplementary Data [file supp_erw165_supplementary_figures_S1_S2.pdf]

***Journal of Experimental Botany* Supporting Information**

Article title: Life without complex I:
Proteome analyses of an Arabidopsis mutant lacking the mitochondrial NADH dehydrogenase complex

Authors: Steffanie Fromm, Jennifer Senkler, Holger Eubel, Christoph Peterhänsel, Hans-Peter Braun

Article acceptance date: 21 March 2016

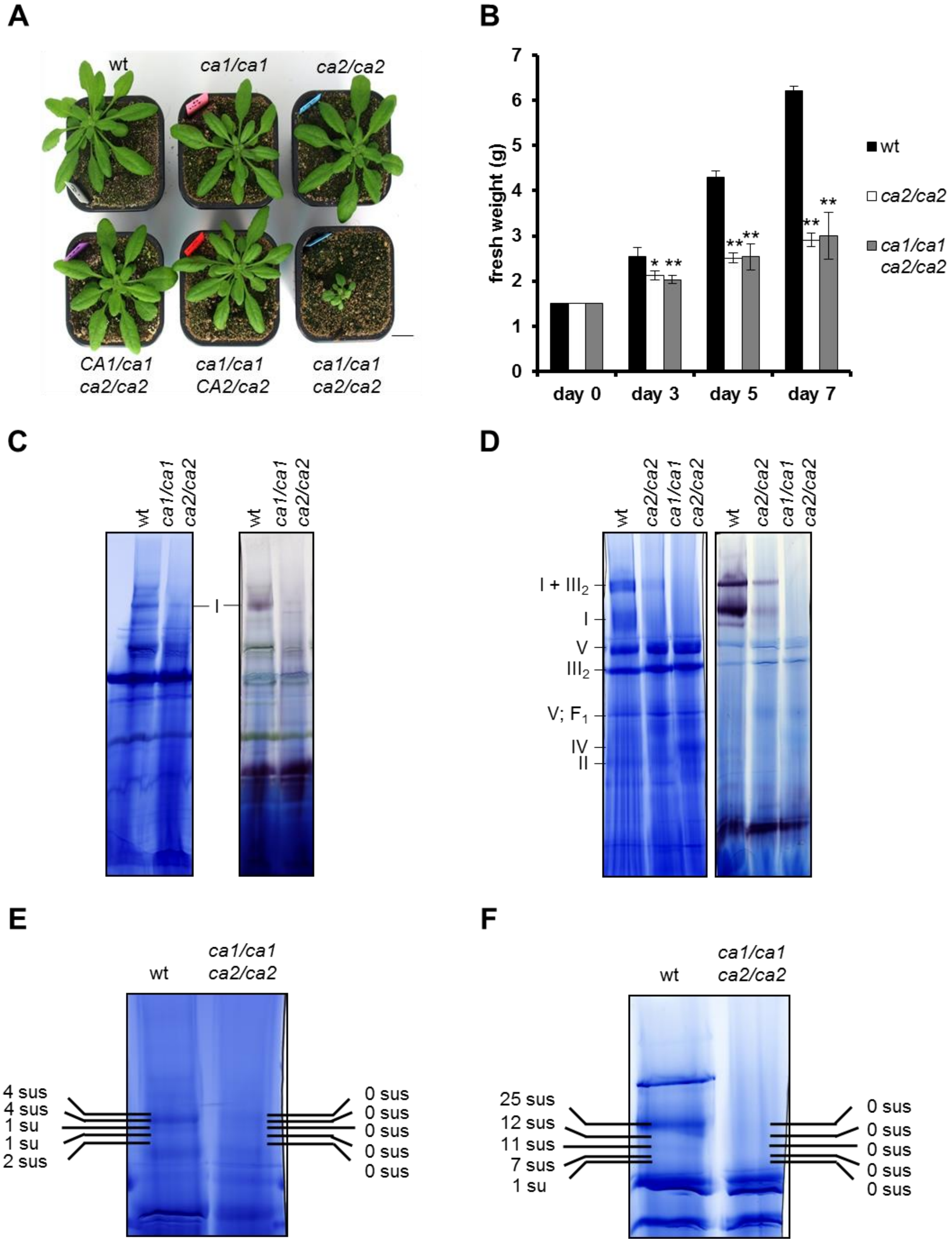
A
wt
ca1/ca1
ca2/ca2
CA1/ca1 ca2/ca2
ca1/ca1 CA2/ca2
ca1/ca1 ca2/ca2
B
fresh weight (g)
0
1
2
3
4
5
6
7
day 0
day 3
day 5
day 7
*
**
wt
ca2/ca2
ca1/ca1 ca2/ca2
C
wt
ca1/ca1 ca2/ca2
I
D
wt
ca2/ca2
ca1/ca1 ca2/ca2
I + III_2
I
V
III_2
V; F_1
IV
II
E
wt
ca1/ca1 ca2/ca2
4 sus
4 sus
1 su
1 su
2 sus
0 sus
0 sus
0 sus
0 sus
0 sus
F
wt
ca1/ca1 ca2/ca2
25 sus
12 sus
11 sus
7 sus
1 su
0 sus
0 sus
0 sus
0 sus
0 sus

Fig. S1 Growth phenotype of *ca1ca2* plants, absence of complex I. A: Rosettes of ten weeks old plants grown under short-day (10 h : 14 h, light : dark) conditions. Scale bar is 2 cm. *ca1/ca1*: plant line homozygous for knock-out of *CA1*; *ca2/ca2*: plant line homozygous for knock-out of *CA2*; *CA1/ca1 ca2/ca2*: plant line homozygous for knock-out of *CA2* but hemizygous for knock-out of *CA1*; *ca1/ca1 CA2/ca2*: plant line homozygous for knock out of *CA1* but hemizygous for knock out in *CA2*; *ca1/ca1 ca2/ca2*: plant line homozygous for knock-outs of *CA1* and *CA2*. B Fresh weight increase of Arabidopsis wt, *ca*2 and *ca1ca2* cell cultures. Starting material (day 0) for wt (black bars), *ca2* (white bars), and *ca1ca2* (grey bars) cell cultures was always 1.5 g. Fresh weight (g) was recorded after three, five and seven days (n = 9, mean ± SE). * = $p \leq 0.05$; ** = $p \leq 0.01$ according to Student's t-test mutants compared to wt. C and D: Protein complexes of mitochondria isolated from leaves (C) and cell culture (D) were resolved by BN-PAGE. Gels were stained with colloidal Coomassie. Corresponding gels were used for *in gel* activity assays of complex I. Identities of selected mitochondrial protein complexes are indicated beside the gels (I: complex I; II: complex II; III_2: dimeric complex III; $I+III_2$: supercomplex formed of complex I and dimeric complex III; IV: complex IV; V: complex V; F1: F1 part of complex V). Note: the faint band at the position of complex I in the mitochondria from leaves of the double mutant does not represent complex I as determined by mass spectrometry (data not shown and data shown in the following figure parts) E and F: Protein complexes of isolated mitochondria of wt and *ca1ca2* of leaves (E) and cell culture (F) were separated by BN-PAGE and Coomassie stained afterwards. In the surrounding of complex I proteins were analyzed by LC-MS. The number of identified complex I subunits (su/sus) for wt and *ca1ca2* mutant is given beside the gels. Some parts of this figure represent modified versions of figures published previously in Fromm *et al.* 2016c.

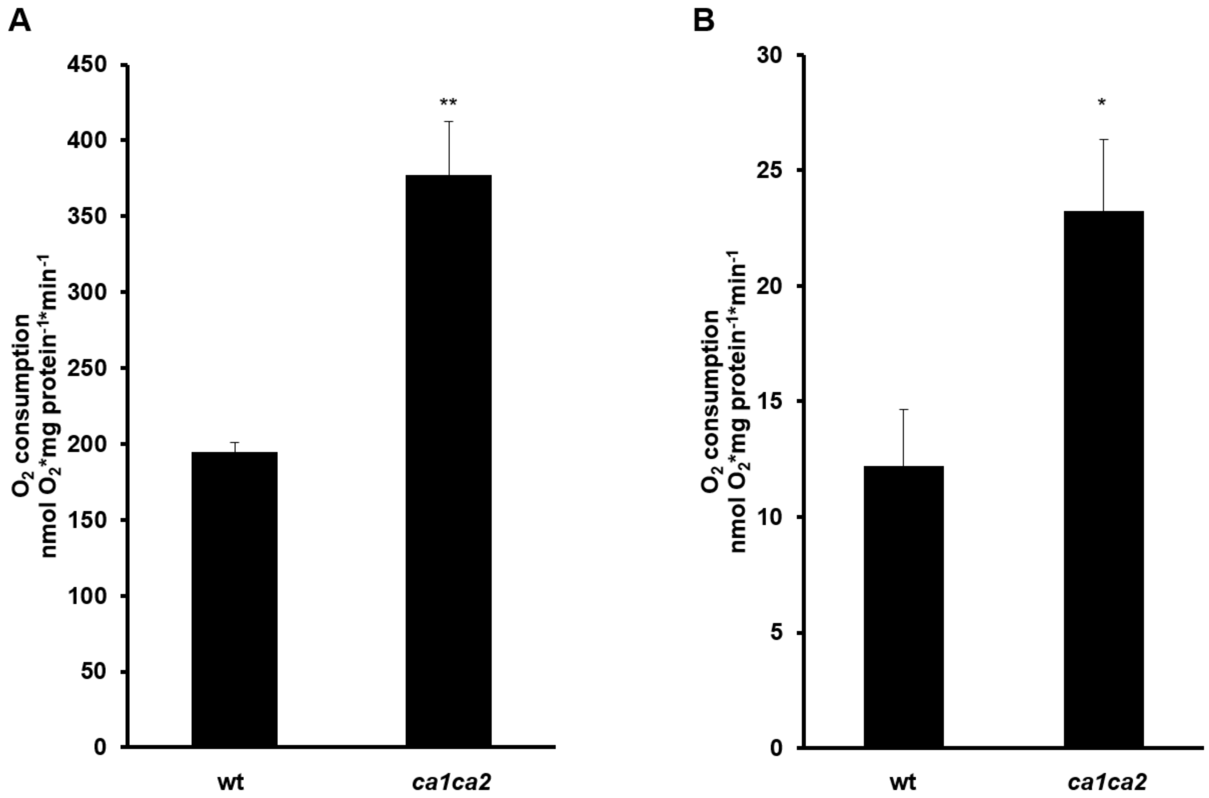

Fig. S2 Respiration through complex II and AOX capacity of mitochondria derived from *Arabidopsis thaliana* wildtype (wt) and *ca1ca2* double mutant lines. Oxygen consumption of isolated mitochondria was measured to estimate the respiration through complex II by adding succinate as substrate (A) and the capacity of AOX by adding AOX inhibitor n-propyl gallate (nPG) (B) using a Clark-type oxygen electrode (n = 5, mean ± SE). * = $p \leq 0.05$; ** = $p \leq 0.01$ according to Student's t-test mutants compared to wt.
